# LAR Receptor Tyrosine Phosphatase Family in Healthy and Diseased Brain

**DOI:** 10.3389/fcell.2021.659951

**Published:** 2021-12-13

**Authors:** Francisca Cornejo, Bastián I. Cortés, Greg M. Findlay, Gonzalo I. Cancino

**Affiliations:** ^1^ Center for Integrative Biology, Facultad de Ciencias, Universidad Mayor, Santiago, Chile; ^2^ MRC Protein Phosphorylation and Ubiquitylation Unit, School of Life Sciences, University of Dundee, Dundee, United Kingdom; ^3^ Escuela de Biotecnología, Facultad de Ciencias, Universidad Mayor, Santiago, Chile

**Keywords:** brain disorders, protein phosphatase, receptor protein tyrosine phosphatase (RPTP), PTPdelta, PTPsigma

## Abstract

Protein phosphatases are major regulators of signal transduction and they are involved in key cellular mechanisms such as proliferation, differentiation, and cell survival. Here we focus on one class of protein phosphatases, the type IIA Receptor-type Protein Tyrosine Phosphatases (RPTPs), or LAR-RPTP subfamily. In the last decade, LAR-RPTPs have been demonstrated to have great importance in neurobiology, from neurodevelopment to brain disorders. In vertebrates, the LAR-RPTP subfamily is composed of three members: PTPRF (LAR), PTPRD (PTPδ) and PTPRS (PTPσ), and all participate in several brain functions. In this review we describe the structure and proteolytic processing of the LAR-RPTP subfamily, their alternative splicing and enzymatic regulation. Also, we review the role of the LAR-RPTP subfamily in neural function such as dendrite and axon growth and guidance, synapse formation and differentiation, their participation in synaptic activity, and in brain development, discussing controversial findings and commenting on the most recent studies in the field. Finally, we discuss the clinical outcomes of LAR-RPTP mutations, which are associated with several brain disorders.

## Introduction

Post-translational regulation involves covalent modifications that control protein activity, with phosphorylation being the most common modification (Tonks, 2006). Since reversible phosphorylation is a major feature in cellular signaling (Cohen, 2002), dephosphorylation reactions are equally important for controlling cellular processes. The complementary roles of protein kinases and protein phosphatases have been underlined by studies showing that protein kinases mediate the amplitude of a signal, whilst protein phosphatases may control its rate and duration (Tonks, 2006). Eukaryotic protein phosphorylation typically occurs on serine, threonine or tyrosine residues and protein phosphatases are often classified according to the residue that they dephosphorylate and/or the homology of their catalytic domain (Liberti et al., 2013; Chen et al., 2017). Protein tyrosine phosphatases are classified in two families; the receptor protein tyrosine phosphatase (RPTP) and the non-receptor tyrosine phosphatases family, which play important roles in intercellular communication and intracellular signal transduction (Cohen, 2002; Chen et al., 2017).

RPTPs were discovered in 1988 (Tonks et al., 1988), and have been increasingly studied because they not only participate in cellular signaling through their phosphatase activity, but also by acting as adhesion molecules often independently of their catalytic domains (Young et al., 2021). Amongst these dual-function molecules, type IIA RPTPs arise as important modulators of several cellular processes within the brain, acting as signaling and adhesion molecules. Type IIA RPTPs, also known as Leukocyte common Antigen-Related RPTP (LAR-RPTP) subfamily, are integral membrane proteins which regulate the activation of several signaling pathways by modulating tyrosine phosphorylation (Tonks, 2006; Coles et al., 2015). Indeed, a phospho-proteomic study in mouse embryonic cells revealed that activity of one member of the LAR-RPTP subfamily, PTPRF (LAR), regulates the phosphorylation state of at least 205 different proteins (Sarhan et al., 2016b). This illustrates the importance of LAR-RPTPs catalytic activity in regulating essential and diverse cellular processes such as protein synthesis and degradation, cytoskeleton organization, cell adhesion and migration, and protein transport among others (Sarhan et al., 2016b).

In the recent years, several studies have shown that LAR-RPTPs have important roles in the regulation of biological processes within the brain, from neural development to synaptic function and differentiation (reviewed in Chagnon et al., 2004; Tonks, 2006; Takahashi and Craig, 2013; Um and Ko, 2013; Stoker, 2015; Han et al., 2016; Won and Kim, 2018), which illustrates the importance of LAR-RPTPs in the regulation of several neural signaling pathways, and the detrimental effects that could induce its impaired expression over a wide number of essential brain processes. Therefore, we will focus on LAR-RPTPs function in the mammalian brain, reviewing the main evidence for their participation in neurobiological processes. We will discuss recent studies that suggest a secondary role for LAR-RPTPs in synapse development in mammals, in contrast to previous studies that assigned them a fundamental role in synaptogenesis. Also, we will summarize LAR-RPTPs participation in the etiology of neurological and psychiatric disorders, highlighting the importance of studying LAR-RPTPs as potential therapeutical targets for brain diseases.

## LAR-RPTP Types and Structure

**FIGURE 1:**
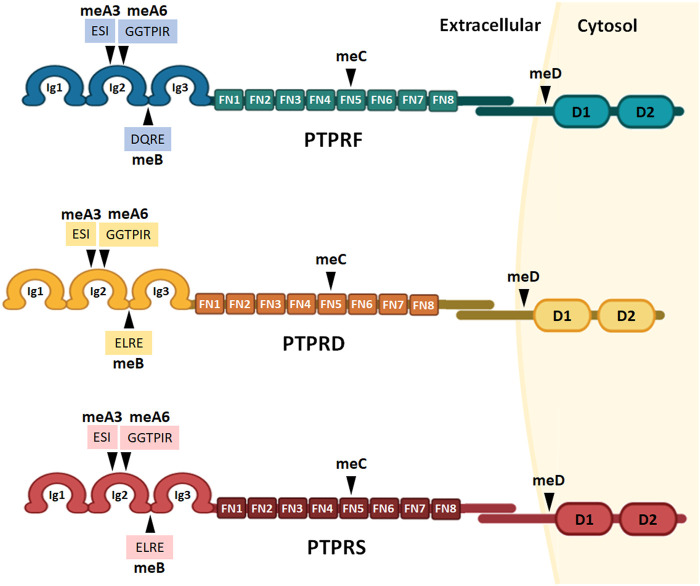
LAR-RPTPs protein structure and alternative splicing sites. The structure of all three receptors consists of an extracellular portion of three Ig-like domains and eight extracellular fibronectin type III domains, a transmembrane region, and two intracellular protein tyrosine phosphatase domains: a membrane proximal catalytically active domain and a membrane distal domain with no phosphatase activity. Alternative splicing sites are marked as mini-exons meA-D, and the aminoacidic sequences of meA3, meA6 and meB are shown. Ig: Ig-like domains; FN: fibronectin type III domains; D1 and D2: phosphatase domains.

The LAR-RPTP subfamily is composed of three members in vertebrate organisms: PTPRF (also known as LAR), PTPRD (PTPδ) and PTPRS (PTPσ). These genes share up to 72% identity in humans (Coles et al., 2015). *PTPRF, PTPRD* and *PTPRS* are located at chromosome 1 p34.2, chromosome 9 p24.1-p23, and chromosome 19 p13.3, respectively, and as discussed in Section 3, all three LAR-RPTPs have multiple isoforms. LAR-RPTPs share a similar structure and domain organization, consisting of three extracellular Ig-like domains, eight extracellular fibronectin type III (FNIII) domains, a transmembrane region, and two intracellular protein tyrosine phosphatase (PTP) domains (Figure 1). In the extracellular region, the three Ig-like domains fold into a V-shaped conformation that is fundamental for LAR-RPTPs ligand binding activity (Kwon et al., 2010; Yamagata et al., 2015). The FNIII domains have a “beads on a string” conformation which is flexible for motility within the synaptic cleft, and to regulate clustering of LAR-RPTPs and their interaction with various ligands (Won and Kim, 2018).

The intracellular region of LAR-RPTPs consists of two PTP domains: a membrane proximal PTP domain (D1) that is catalytically active due to a cysteine residue that is required for substrate tyrosine dephosphorylation, and a membrane distal domain (D2) that has no catalytic activity but is important for stabilizing LAR-RPTPs in the synaptic zone (Chagnon et al., 2004). This function is fulfilled via binding to intracellular molecules, and by acting as a docking domain for the interaction with other receptors and scaffolding proteins such as liprins to modulate synapse formation (Streuli et al., 1990; Pulido et al., 1995; Dunah et al., 2005). Among the three LAR-RPTPs, D2 domains are more highly conserved than D1 (Krueger et al., 1990), and despite the lack of catalytic activity, in the case of PTPRF, replacement of two specific residues (Leu-1644-to-Tyr and Glu-1779-to-Asp) can restore phosphatase activity to the D2 domain (Nam et al., 1999). Therefore, the different domains of LAR-RPTPs confer them the particularity of acting as adhesion molecules and ligand receptors in the extracellular, and as phosphatases and scaffolding proteins in the intracellular, which illustrates the diversity of molecular functions of these proteins in the cell.

## LAR-RPTP Isoforms and Alternative Splicing

During neuronal development, alternative splicing events are precisely coordinated by the combinatorial effects of RNA-binding proteins, leading to neuron-specific splicing isoforms (Weyn-Vanhentenryck et al., 2018). In the case of LAR-RPTPs, alternative splicing regulates the inclusion of four mini-exons (short peptide sequences of up to 16 amino acids; meA-D, Figure 1). LAR-RPTPs mini-exon peptide sequences are encoded by micro-exons (nucleotide sequences of shorter than 30 nucleotides), which are part of a highly conserved and dynamic network where micro-exons are critical for neuronal alternative splicing events (Irimia et al., 2014; Li et al., 2015; Parada et al., 2021).

The inclusion of mini-exon meA is determined by the alternative splicing of two micro-exons, which yield three possible meA variants; meA3 (ESI), meA6 (GGTPIR) and their tandem combination meA9 (ESIGGTPIR), which are inserted in the Ig2 domain (Figure 1). The meB peptide sequence is encoded by a single micro-exon, which incorporates either a DQRE for PTPRF, or ELRE residues for PTPRS and PTPRD. In each case, the meB peptide is inserted between the Ig2 and Ig3 domains (Figure 1) (Yamagata et al., 2015; Yoshida et al., 2021). Mini-exons meC and meD are inserted in the FN5 domain and near the D1 catalytic domain respectively (Figure 1).

While the biological functions of meC and meD have not yet been described, meA and meB inclusions have fundamental roles in modulating LAR-RPTPs ligand binding activity. Therefore LAR-RPTPs alternative splicing contributes to a molecular code of the synaptic organization. Alternative splicing at meA and meB sites is key to determining the Ig-mediated binding affinities for a wide range of synaptic proteins, including TrkC, IL1RAPL1, IL1RAcP, Slitrks, SALMs, and Neurexins (Han et al., 2016; Han et al., 2020a, Li et al., 2015; Takahashi et al., 2011; Yoshida et al., 2011, 2012). For example, only the PTPRD isoforms that contain meA9 or meA6 can bind to IL1RAPL1. Although the meA6 variant requires the inclusion of meB to interact with IL1RAPL1, meA9 insertion alone is sufficient to enable PTPRD/IL1RAPL1 interaction (Yamagata et al., 2015). Also, since meA inserts on PTPRD only partially interact with IL-1RAcP, the strength of PTPRD/IL-1RAcP interaction is mostly determined by meB (Han et al., 2016; Won and Kim, 2018). Although meB inclusion represents a subtle amino acid change of the LAR-RPTPs protein sequences, the functional consequences are profound since the meB insertion introduces a flexible linker between the Ig2 and Ig3 domains. In the case of PTPRD, the linker enables the interactions with IL-1RAcP, IL1RAPL1, and SALM3, and for PTPRS, it enables interaction with Slitrk and inhibits the interaction with TrkC (Han et al., 2016; Won and Kim, 2018).

The molecular mechanisms that regulate the expression of different LAR-RPTP isoforms are unknown. However, it can be hypothesized that a complex signaling network tightly regulates their alternative splicing, as it modulates LAR-RPTPs coupling to specific ligands and synaptic partners to drive fundamental neurodevelopmental processes such as synaptic differentiation (reviewed in Fukai and Yoshida, 2020; Han et al., 2016; Takahashi and Craig, 2013; Um and Ko, 2013). Therefore, the different LAR-RPTP isoforms generated by micro-exons alternative splicing might participate in different cellular processes, which highlights the importance of understanding the biological functions of each alternative splicing variant, especially those generated by the inclusion of meD, since given its proximity to the catalytic domain, it could regulate substrate affinity of the LAR-RPTPs.

## Processing and Regulation of LAR-RPTPs Activity

**FIGURE 2:**
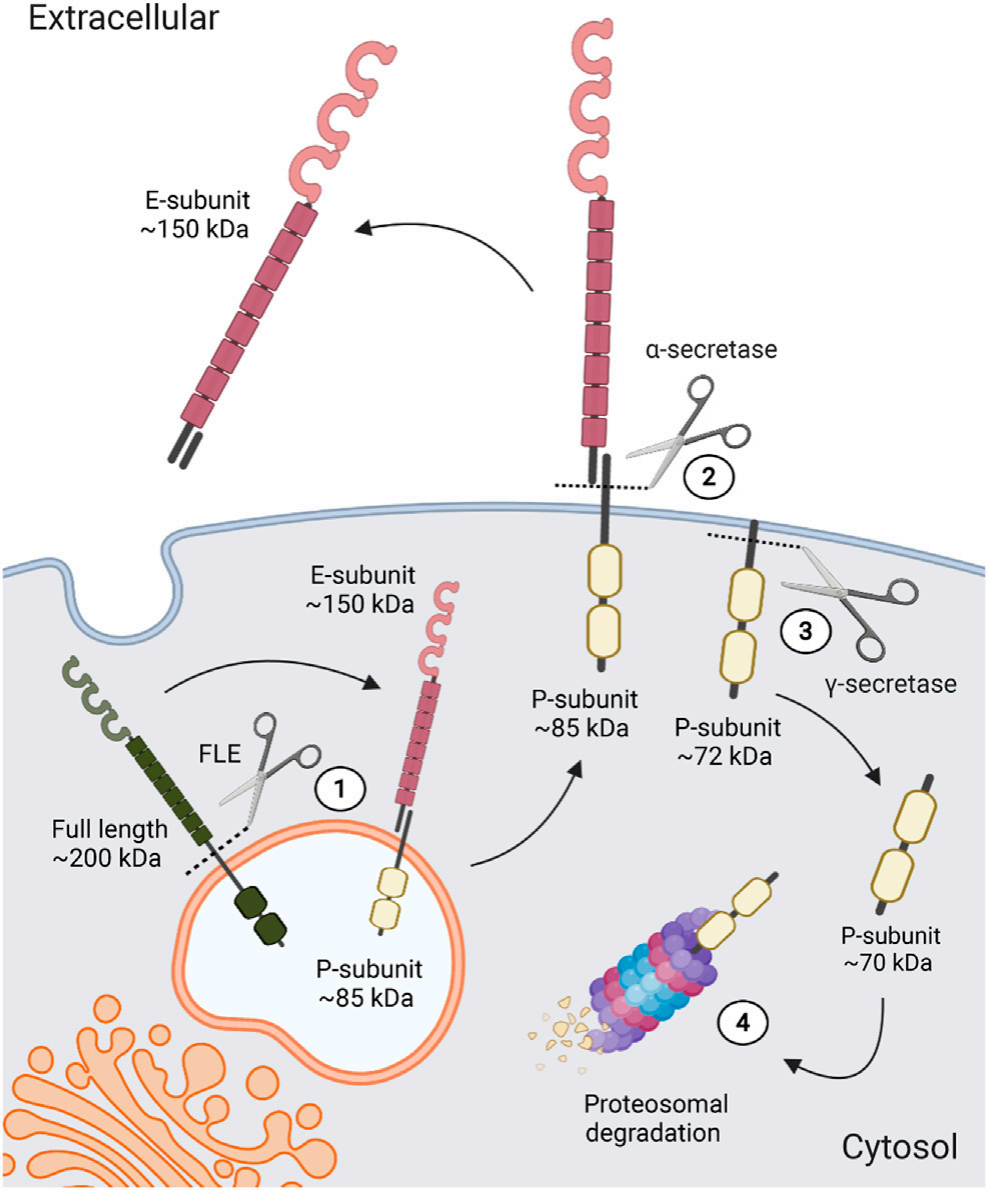
LAR-RPTPs proteolytic processing. After its translation, LAR-RPTPs are processed in the trans-Golgi by furin-like endoproteases (FLE) **(1)**, to later be translocated to the cell surface, where they will be integrated into the membrane as a complex of two subunits; the extracellular E-subunit and the intracellular P-subunit, who remain non-covalently bound. Extracellularly, α-secretase can also induce a cleavage in the ectodomain of the P-subunit **(2)**, which releases the extracellular portion of LAR-RPTPs. Also, intracellular tandem phosphatase domains are proteolytically processed by γ-secretase **(3)**, inducing LAR-RPTPs catalytic region internalization and its proteasomal degradation **(4)**.

LAR-RPTPs are expressed as a ∼200-KDa protein that can undergo proteolytic processing mediated by furin-like endoproteases in the trans-Golgi to be translocated to the cell surface (Figure 2) (Aicher et al., 1997; Serra-Pagès et al., 1994; Streuli et al., 1992). These endoproteases recognize a penta-arginine sequence at the C-terminus of the extracellular domain which generates a 150-KDa extracellular subunit (E-subunit) that remains noncovalently bound to a 85-KDa subunit (P-subunit) containing a short ectodomain, the transmembrane peptide, and the two intracellular phosphatase domains (Serra-Pagès et al., 1994). LAR-RPTPs can also undergo a second α-secretase-dependent proteolytic processing at a site within the P-subunit ectodomain near the transmembrane region, which releases the extracellular region from the cell surface and promotes P-subunit internalization (Haapasalo et al., 2007). Mature LAR-RPTPs can also undergo proteolytic processing of the intracellular tandem phosphatase domains, which appears to play an important role in regulating LAR-RPTPs intracellular signaling. It has been observed that LAR-RPTPs catalytic activity is down-regulated by γ-secretase mediated proteolytic processing, which induces the internalization of the catalytic region for its proteasomal degradation, thereby reducing their intracellular signaling (Figure 2) (Aicher et al., 1997; Haapasalo et al., 2007; Coles et al., 2015). Alternatively, it has been proposed that the internalized LAR-RPTP catalytic region could regulate transcription, as PTPRF intracellular fragment cleaved by γ-secretase enter the nucleus and interact with β-catenin, dephosphorylating it and reducing its transcriptional activity (Haapasalo et al., 2007).

LAR-RPTPs cleaved E-subunit have been mostly observed in cell cultures media (Craig and Brady-Kalnay, 2011), suggesting that all three LAR-RPTPs are shed and might be exerting extracellular signaling through the released fragment. In the rat brain, it has been shown that a PTPRF short ectodomain (a segment of the fifth FNIII domain) forms a homophilic interaction with mature PTPRF to regulate neurite outgrowth (Yang et al., 2003). Similarly, PTPRD homophilic interaction with its extracellular fragment has been shown to promote axonal growth (Sun et al., 2000). Finally, although PTPRS homophilic interaction has not yet been documented, PTPRS ectodomains have been shown to promote neurite outgrowth non-cell-autonomously (Sajnani, et al., 2005), suggesting that LAR-RPTPs cleaved extracellular domains act as paracrine signaling ligands that drive the growth of neuronal structures. More importantly, as LAR-RPTPs interact with several ligands to regulate neurobiological processes, cleaved ectodomains may antagonize mature LAR-RPTP interaction with their *trans*-synaptic ligands. Therefore, extracellular proteolytic processing could be a negative-feedback mechanism for LAR-RPTPs *trans*-synaptic signaling.

As mentioned before, the D2 domain lacks PTP activity as a result of a substitution of the critical residues that recognize the substrate phosphotyrosine, which initially suggested that the D2 domain played no role in catalysis (Streuli et al., 1990; Nam et al., 1999; Won and Kim, 2018). However, it has been shown that the D2 domain has an important function modulating D1 catalytic activity by regulating the substrate specificity (Streuli et al., 1990), and by participating in LAR-RPTP inhibition. It has been shown that heterodimerization of the PTPRD-D2 domain and the PTPRS-D1 domain negatively regulates catalytic activity of PTPRS without affecting the activity of PTPRD (Wallace et al., 1998). Also, it has been observed that LAR-RPTPs homophilic D1/D1 interaction decreases their phosphatase activity by hindering the substrate-binding pocket in the D1 domain (Coles et al., 2011; Xie et al., 2020), suggesting that LAR-RPTPs negatively regulate their phosphatase activity by forming dimers at the plasma membrane.

LAR-RPTPs homophilic or heterophilic interaction is mediated by a helix-loop-helix (HLH), wedge-shaped motif located between the membrane proximal region and the D1 domain, which mediates catalytic inhibition. The use of LAR-RPTP wedge peptides have been proved to successfully inhibit LAR-RPTPs functions in brain cells (Xie et al., 2006; Lang et al., 2015). Interestingly, in some cases LAR-RPTP wedge peptides can modulate their extracellular ligand binding activity. For example, in neuronal cell cultures, the treatment with peptide-mimetics of the PTPRS wedge motif decreases the extracellular interaction of PTPRS with chondroitin sulfate proteoglycans (CSPGs) ligands (Lang et al., 2015), which indicates that LAR-RPTPs catalytic inhibition could induce conformational changes that modify ligand affinity.

It has also been observed that some LAR-RPTPs catalytic activity is modulated by oxidation, where the oxidative state of D1 and D2 domains determines LAR-RPTP phosphatase activity (Groen et al., 2005). PTPRS catalytic activity is reduced in cells exposed to UV through an unknown mechanism that oxidizes a cysteine in its active site *in vitro* (Groß et al., 1999). Also, it has been observed that oxidation induces PTPRF conformational changes in D1 and D2 domains, which promotes its dimerization (Groen et al., 2008), suggesting another mechanism for regulating LAR-RPTP catalytic activity. However, it remains to be determined how the microenvironment (pH and UV radiation) and the oxidative state of LAR-RPTPs modulates their catalytic activity.

LAR-RPTPs proteolytic processing appears to be important to regulate their signaling, although more studies are required to determine the physiological function of the different cleaved domains, especially *in vivo*. Also, their catalytic activity is tightly regulated by dimerization, which is mediated by the interaction between intracellular domains, suggesting an important role for LAR-RPTPs membrane clustering. However, the cellular mechanisms that promote dimerization of the LAR-RPTPs remain largely understudied, as well as the molecular changes induced by microenvironmental factors that modulate their catalytic activity.

## LAR-RPTPs in Neurite Growth and Axon Guidance

In the last few decades, LAR-RPTPs have been shown to have important roles in several signaling pathways in the brain, such as in the regulation of dendrite and axon growth. LAR-RPTPs mediate cell-cell or cell-extracellular matrix adhesion to promote neurite outgrowth, and these processes often depends on the binding of LAR-RPTPs to CSPGs and heparan sulfate proteoglycans (HSPGs) (Johnson et al., 2006; Fisher et al., 2011; Chien and Ryu, 2013). The first *in vitro* demonstration that all three LAR-RPTPs participate in neurite outgrowth was carried out in retinal and hippocampal neurons, and PC12 cells (Tisi et al., 2000; Yang et al., 2003; Yang et al., 2005), which was confirmed later in studies using knockout mice and LAR-RPTPs blocking peptides (Fisher et al., 2011; Chien and Ryu, 2013). The intracellular pathways involved in axonal guidance have largely been described in invertebrate models such as *Caenorhabditis elegans* and *Drosophila melanogaster*. A major signaling effector is Trio, a guanine nucleotide exchange factor (GEF) for Rac1 and RhoA, which interacts with the D2 domain of LAR-RPTPs to promote axon guidance (Debant et al., 1996; Ball et al., 2010; Fuentes-Medel and Budnik, 2010). Trio also binds to ABL1 (also known as c-Abl) and Ena/VASP to regulate axon growth, in a mechanism that requires its direct interaction with Dlar, a *Drosophila* LAR-RPTP ortholog (Wills et al., 1999). The intracellular signaling mediated by Trio, ABL1 and Dlar is required for actin cytoskeleton remodeling associated with axon growth (Um and Ko, 2013). LAR-RPTPs also mediate motor axon guidance signaling via direct interaction with calcium/calmodulin-dependent serine protein kinase-interacting proteins (Caskin 1 and Caskin 2), which form a signaling complex for axon growth and guidance (Weng et al., 2011).

### PTPRF

A predominant function of PTPRF is the regulation of the actin cytoskeleton (Sarhan et al., 2016b). It has been observed that PTPRF knockout leads to a reduction in the number of focal adhesions and reduced adhesion to the extracellular matrix, suggesting PTPRF as a part of a complex that links actin cytoskeleton to the extracellular matrix and forms focal adhesions to promote neurite growth (Dunah et al., 2005; Sarhan et al., 2016a). PTPRF interaction with extracellular matrix ligands promote its catalytic activation and the dephosphorylation the tyrosine kinase ABL1, which promotes AKT and CDK1 activation (Sarhan et al., 2016a), increasing cell adhesion to extracellular matrix and favoring the growth of dendrites and axons (Serra-Pagès et al., 1995). Also, PTPRF homophilic interaction with its ectodomain has been shown to promote neurite outgrowth, in a mechanism where the ectodomain acts as a ligand to induce PTPRF phosphatase activity, which then participates in the intracellular activation of several signaling pathways (Yang et al., 2003, 2005).

### PTPRD

A role for PTPRD in dendrite growth was discovered in PTPRD knockout mice, which display reduced dendritic branching, length, and thickness (Nakamura et al., 2017). PTPRD promotes dendrite growth by dephosphorylating and activating Fyn and Src kinases, which induces the arborization of dendrites mediated by Semaphorin-3A (Nakamura et al., 2017). Also, Semaphorin-3A-induced growth cone collapse response has been shown to be dependent on PTPRD expression, suggesting direct participation of PTPRD in axon growth (Nakamura et al., 2017)*.* Besides, a soluble gradient of PTPRD induces chemoattraction of growth cones in neuronal cultures, in a mechanism dependent on tyrosine phosphatase activity (Sun et al., 2000), which highlights the dual role of PTPRD as a ligand and as a signaling molecule in axon growth regulation. However, PTPRD knockout mice do not show gross impairment in axon growth, while knocking out both PTPRD and PTPRS induces axon degeneration as peripheral nerves fail to contact their targets (Uetani et al., 2006). This indicates that LAR-RPTPs may have redundant roles in axon growth regulation (Stoker, 2015).

### PTPRS

**FIGURE 3:**
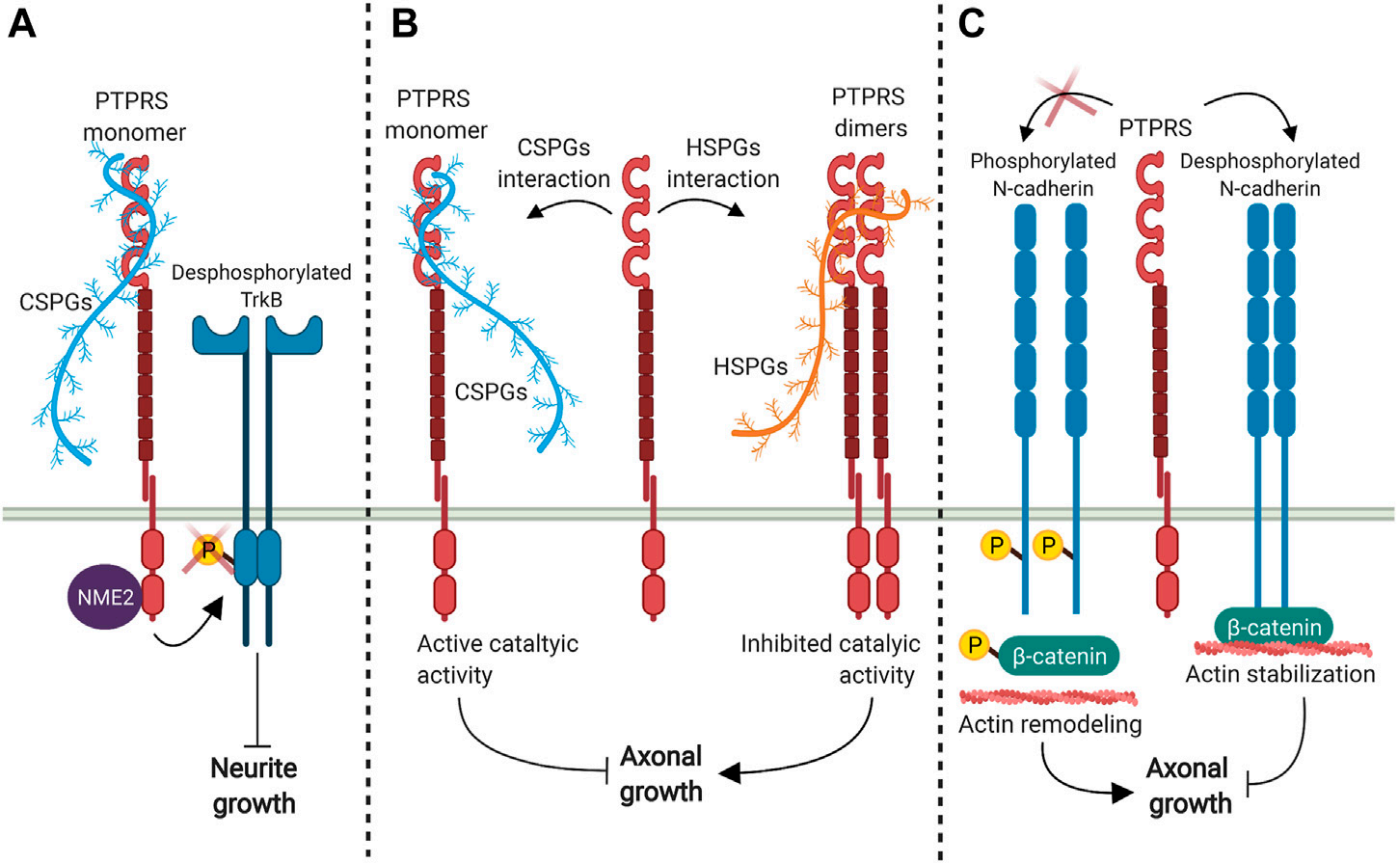
PTPRS signaling modulates dendrite and axon growth. **(A)** PTPRS interaction with CSPGs promotes TrkB dephosphorylation which reduces dendrite growth in a mechanism that appears to be mediated by PTPRS-NME2 interaction (Kurihara and Yamashita, 2012; Lesnikova et al., 2020). **(B)** PTPRS-HSPGs interaction induce PTPRS dimer formation, which inactivates its catalytic activity and favors axon growth; while the interaction with CSPGs promotes the PTPRS monomer conformation, inducing its catalytic activity and inhibits axonal growth (Shen et al., 2009; Coles et al., 2011). **(C)** PTPRS interaction with N-cadherin dephosphorylates N-cadherin and *β*-catenin, which favors N-cadherin-*β*-catenin interaction, stabilizes actin cytoskeleton, and reduces axonal growth (Siu et al., 2007).

PTPRS has the opposite role in the development of dendrites compared to PTPRF and PTPRD, as PTPRS knockout mice show increased dendritic length *in vivo* (Horn et al., 2012). The inhibition of dendritic formation mediated by PTPRS depends on its direct interaction with its ligand CSPG, which induces PTPRS-mediated TrkB dephosphorylation, thereby suppressing dendritic spine growth (Figure 3A) (Kurihara and Yamashita, 2012; Lesnikova et al., 2020). Neurite outgrowth inhibition induced by CSPGs also appears to be mediated by the intracellular interaction between PTPRS and the nucleoside diphosphate kinase 2 NME2 (Figure 3A) (Hamasaki et al., 2016).

PTPRS also negatively regulates axon growth, as PTPRS knockout or catalytic inactivation has been widely reported to activate axonal elongation. Interaction with HSPGs ligands induces the formation of PTPRS dimers, which inactivates its phosphatase activity and promotes axon elongation (Figure 3B), while CSPGs engagement promotes the PTPRS monomer conformation, unleashing its catalytic activity and inhibiting growth cone elongation (Coles et al., 2011; Lang, et al., 2015; Shen et al., 2009). Furthermore, embryonic cortical neurons isolated from PTPRS knockout animals showed an increased rate of axonal elongation (Thompson et al., 2003). PTPRS deficient animals also showed significantly accelerated axonal regeneration in facial motor neuron axotomy, sciatic nerve crush injury and spinal hemisection models (McLean et al., 2002; Thompson et al., 2003; Fry et al., 2009). The same results were observed when axonal regeneration was evaluated after optic nerve injury, where the number of axons that cross the lesion site was higher in PTPRS deficient mice (Sapieha et al., 2005). Besides, mice lacking PTPRS also show increased axon collateral branching in the hippocampus during normal aging or following chemically induced seizure (Horn et al., 2012), suggesting that PTPRS has an important role in maintaining neuronal structures by suppressing dendritic formation, and axonal growth and branching. Also, catalytic inhibition of PTPRS using a wedge motif peptide-mimetic efficiently restores axonal elongation in mice models of spinal cord injury, recovering the serotoninergic innervation into the spinal cord (Lang et al., 2015). Therefore, PTPRS may be a promising therapeutic target for axonal degeneration pathologies.

N-cadherin and *β*-catenin have been proposed as the substrates that mediates PTPRS participation in the inhibition of axonal growth. The dephosphorylation of N-cadherin and *β*-catenin by PTPRS promotes N-cadherin-*β*-catenin complex formation, which favors the association between N-cadherin and the actin cytoskeleton to reduce axonal growth (Figure 3C) (Siu et al., 2007). Another substrate proposed to interact with PTPRS is p250GAP (Chagnon et al., 2010), a GTPase-activating protein that regulate the small GTPases RhoA, Rac and Cdc42 (Moon and Zheng, 2003; Nakazawa et al., 2003). This protein is widely expressed in the embryonic and adult brain with an expression pattern similar to PTPRS (Schaapveld et al., 1998; Simó and Cooper, 2012). Neuronal cultures obtained from p250GAP deficient animals presented longer neurites compared to wild-type mice (Simó and Cooper, 2012), a phenotype similar to PTPRS-deficient neuronal cultures (Thompson et al., 2003), suggesting that p250GAP and PTPRS could be acting in the same pathway. As the activity of p250GAP increases in the presence of PTPRS (Chagnon et al., 2010), and its activity is inhibited by phosphorylation (Okabe et al., 2003), p250GAP dephosphorylation and activation mediated by PTPRS could therefore be important to restrict neurite outgrowth.

The participation of LAR-RPTPs in several signaling pathways that regulate neurite outgrowth and axon guidance reveals important roles of LAR-RPTPs to ensure correct brain development. However, a recent paper has disputed the participation of LAR-RPTPs in the regulation of neuronal morphology. Sclip and Südhof (2020) have found that knocking out LAR-RPTP expression after neurogenesis but before synapse formation do not affect dendritic and axonal growth, which was observed when the genes encoding all three LAR-RPTPs, singly or in combination, were deleted in cultured neurons, suggesting that LAR-RPTPs expression is expendable for neuronal development, at least in hippocampal neurons (Sclip and Südhof, 2020). These results, which contradict the evidence summarized above, suggest that functions of LAR-RPTPs in neuronal development needs to be revisited, and highlights the importance of studying the role of LAR-RPTPs in the brain considering factors such as cellular context and developmental stage (Tomita et al., 2020).

## LAR-RPTPs in Synaptic Formation and Function

The first evidence that suggested a role for LAR RPTPs in synapse formation came from their synaptic localization, which was demonstrated by immunofluorescence and co-immunoprecipitation with synaptic proteins such as β-catenin (Kypta et al., 1996; Wyszynski et al., 2002; Dunah et al., 2005; Um and Ko, 2013). β-catenin interacts with N-cadherin to regulate dendritic spine morphogenesis and neurotransmitter vesicles release (Brigidi and Bamji, 2011; Um and Ko, 2013). As BDNF promotes synapse formation by inducing β-catenin phosphorylation, which reduces β-catenin-cadherin interaction (Bamji et al., 2006), it has been suggested that the direct dephosphorylation of β-catenin by LAR-RPTPs could downregulate synapse formation (Um and Ko, 2013). Although direct participation of LAR-RPTPs in β-catenin induced synaptic formation has not been demonstrated, there are numerous studies showing that LAR-RPTPs play fundamental roles in synapse formation though *trans*-synaptic interactions with key synaptic regulators such as SALM3, TrkC, Slitrks, NGL-3 and IL-1RAcP (Woo et al., 2009; Takahashi et al., 2011, 2012; Yoshida et al., 2012; Um et al., 2014; Li et al., 2015). As discussed later, these interactions often induce specific synaptic differentiation.

Recent literature has provided apparently conflicting observations regarding the role of LAR-RPTPs in synapse formation, probably due to the differential experimental approaches that have been carried out. While RNAi-mediated knockdowns of LAR-RPTPs in cell cultures have shown deficiencies in synaptic formation and neurotransmitter release (Dunah et al., 2005; Ko et al., 2015; Han et al., 2018), recent studies using conditional knockout animals have suggested that PTPRD and PTPRS are not essential for synaptic formation at least in the hippocampus (Han et al., 2020c; Sclip and Südhof, 2020). Despite the clear difference between both experimental models, it is important to highlight that other studies using LAR-RPTP knockout mice have shown impairments in behaviors such as spatial learning, memory, motor control and non-REM sleep as discussed later (Wallace et al., 1999; Uetani et al., 2000; Meathrel et al., 2002; Kolkman et al., 2004; Park et al., 2020). Therefore, the different phenotypes observed in constitutive knockout animals, versus mice lacking LAR-RPTPs in specific neural progenitor cells such as Nestin or Emx1 expressing cells, could induce genetic compensation and a “masked” phenotype (Morrison and Münzberg, 2012; Luo et al., 2020). Additional studies using other Cre-driver genes should be evaluated to determine the different cellular mechanisms impaired in each animal model.

Even though the three LAR-RPTPs are mainly expressed presynaptically, where they participate in synaptic differentiation, their expression has been observed postsynaptically at excitatory synapses (Wyszynski et al., 2002; Dunah et al., 2005). It has been suggested that postsynaptic LAR-RPTPs participate in receptor trafficking, reducing the density of AMPA receptors (AMPAR) in hippocampal synapse (Ko et al., 2003a; Dunah et al., 2005; Brigidi and Bamji, 2011). Likewise, LAR-RPTPs regulate AMPAR synaptic transmission through a mechanism mediated by LAR-RPTPs and SALM5 interaction, which promotes the dephosphorylation of AMPAR subunits (Choi et al., 2016). This induces AMPAR internalization and promotes long term depression (LTD) (Dunah et al., 2005; Dickinson et al., 2009). Also, it has been observed that all three LAR-RPTPs control synapse properties by regulating NMDAR-mediated responses, and thus have a critical role in synaptic transmission (Sclip and Südhof, 2020).

### PTPRF

Presynaptic PTPRF participates indirectly in LTD through its interaction with netrin-G ligand-3 (NGL-3) when promotes synaptic differentiation (Woo et al., 2009; Kwon et al., 2010). Treatment with NMDA in cultured neurons or low-frequency stimulation in brain slices induces the proteolytic cleavage of NGL-3, which disrupts the *trans*-synaptic interaction between NGL-3 and PTPRF, impairing synaptic adhesion during LTD, and weakening excitatory synapses (Lee et al., 2014).

### PTPRD

PTPRD knockout mice show enhanced long-term potentiation (LTP) in hippocampal synapses, possibly due to increased neurotransmitter release in the CA1 region. This induces behavioral alterations such as impaired spatial learning and memory, and motor deficits (Uetani et al., 2000), illustrating the importance of PTPRD for hippocampal LTP formation. However, it has also been shown that PTPRD knockout mice have impaired locomotive behaviors and motor weakness, as well as a transient delay in myelination at early postnatal development (Drgonova and Walther, 2015; Zhu et al., 2015), which could partially explain their altered behavior observed in learning tests. PTPRD knockout mice also have impaired synaptic development and decreased excitatory synaptic transmission mainly due to the dysfunction in its interaction with IL1RAPL1 (Park et al., 2020), which highlights the role of PTPRD not only in synaptic formation, but also in excitatory neurotransmission. In contrast, a recent study using pan-neuronal PTPRD conditional knockout animals, showed that PTPRD is not essential for maintenance of excitatory or inhibitory synaptic transmission, since absence of PTPRD did not modify synaptic parameters such as the number of excitatory or inhibitory synapses, miniature excitatory postsynaptic currents (mEPSC), synaptic vesicle tethering, active zone modifications nor neurotransmitter release (Han et al., 2020c), suggesting that PTPRD does not play a significant role in these neurobiological processes. It is important to highlight that Park et al. (2020) found that interaction between PTPRD and IL1RAPL1 is dependent on PTPRD alternative splicing. As much of the signaling mediated by PTPRD depends on interaction with specific ligands, different alternative splicing variants might participate in different cellular processes. Therefore, the controversial results observed in recent studies should be analyzed cautiously, as different alternatively spliced isoforms of PTPRD could generate different neuronal outcomes.

### PTPRS

In presynaptic terminals, PTPRS has been shown to participate in excitatory synaptic function by regulating the localization and size of excitatory synaptic vesicles, modulating glutamate release in the hippocampus, and it also seems to regulate structural features of the active zone such as its length, suggesting that PTPRS participates in the molecular organization of the active zone machinery to efficiently promote glutamate release (Han et al., 2020b). PTPRS is also implicated in regulation of the postsynaptic excitatory neurotransmission. PTPRS interacts with GPC-4 and LRRTM4 to regulate frequency and amplitude of excitatory synaptic transmission. Studies in cultured hippocampal neurons showed that PTPRS knockdown decreased the frequency and amplitude of mEPSC, an effect that was reversed by re-expression of wild-type PTPRS, but not by heparan sulfate (HS)-binding-defective PTPRS mutant that impairs its interaction with GPC-4 and LRRTM4. These observations suggest that PTPRS-GPC-4-LRRTM4 interaction has an important role in the maintenance and function of excitatory synapses (Ko et al., 2015). However, these results contradict previous findings, where mice lacking PTPRS showed increased frequency of mEPSC, which resulted in reduced LTP, and greater paired-pulse facilitation (Horn et al., 2012). One possible explanation could be differential roles for PTPRS splice variants. As in the case of PTPRD, the different synaptic functions in which PTPRS participates must be carefully studied considering the different splicing isoforms, as the interaction of PTPRS with its ligands also depends on alternative splicing (Han et al., 2020a). Recent studies have proposed possible mechanisms by which PTPRS regulates excitatory synapses in the hippocampus (Kim et al., 2020; Sclip and Südhof, 2020). These studies strongly suggest that presynaptic PTPRS promotes LTP through its regulation of NMDAR in the postsynaptic. This is supported by results showing that postsynaptic deletion of PTPRS or the deletion of its extracellular regions required for *trans*-synaptic adhesions do not affect LTP, and therefore PTPRS ligand binding activity would be expendable for NMDAR regulation (Kim et al., 2020). It was also observed that PTPRS catalytic activity mediates dephosphorylation of presynaptic Neurexin-1, which interacts with PTPRS though a complex with liprin-α among others (Serra-Pagès et al., 1995; Hata et al., 1996; Olsen et al., 2005; Weng et al., 2011; Bomkamp et al., 2019), to promote NMDAR-mediated postsynaptic activity (Dai et al., 2019; Kim et al., 2020). These results suggest a mechanism mediated by PTPRS-neurexin-1 presynaptic intracellular complex that regulates NMDAR postsynaptic activation and LTP induction, which is important for recognition memory and novelty preference (Kim et al., 2020). This finding correlates with results obtained by Sclip and Südhof (2020), where a triple conditional knockout mouse for all three LAR-RPTPs show a decrease in NMDAR-mEPSCs in the hippocampus, which is due to a reduction in NMDAR postsynaptic location and not to NMDAR protein levels (Sclip and Südhof, 2020).

The evidence summarized here shows that LAR-RPTP interactions with *trans*-synaptic ligands and with their intracellular substrates modulates a series of synaptic processes required for neuronal function. Even though recent papers have questioned LAR-RPTP participation in some of these biological processes, several evidence showing a primary role in synaptic formation suggest that LAR-RPTP functions can not be underestimated, and more studies are needed to unravel the differential roles that LAR-RPTP alternative splicing variants might have over these neuronal functions.

## LAR-RPTPs in Synapse Differentiation

**FIGURE 4:**
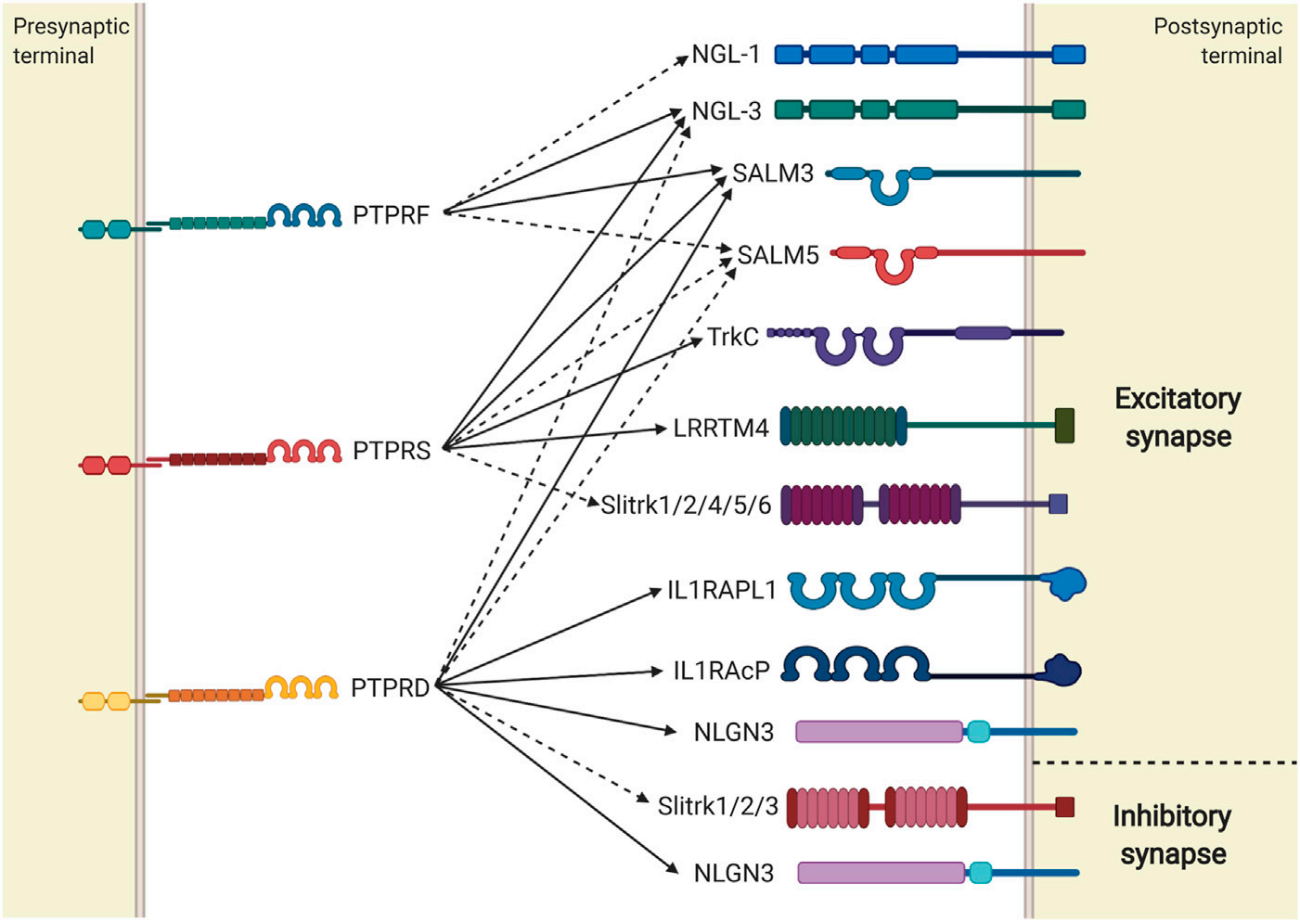
LAR-RPTPs *trans*-synaptic interactions induce synaptic differentiation. Summary of LAR-RPTPs and their synaptic partners whose interactions induce excitatory or inhibitory synapse differentiation. LAR-RPTPs interactions that induce differentiation unidirectionally are represented with dashed lines, while interactions inducing bidirectional differentiation are represented with solid lines.

It is well known that the main roles of LAR-RPTPs at the synapse are as adhesion molecules and as mediators of synapse differentiation. LAR-RPTPs induce presynaptic differentiation by participating in recruitment of synaptic vesicles and the release/recycling machinery, and in some cases, they can also induce postsynaptic differentiation by recruiting neurotransmitter receptors and scaffolding and signaling proteins (reviewed in Takahashi and Craig, 2013). LAR-RPTPs are often located presynaptically at the axon, where they modulate excitatory or inhibitory differentiation depending on their *trans*-synaptic interaction partner (Figure 4). The major *trans*-synaptic partners for each LAR-RPTP have been described, and their interaction determines whether the synapse will be excitatory or inhibitory (Fukai and Yoshida, 2020; Han et al., 2016; Takahashi and Craig, 2013; Um and Ko, 2013). Also, LAR-RPTP interaction with specific partners can shape synaptic differentiation either in a unidirectional manner, inducing differentiation pre-synaptically only, or in a bidirectional way, where this interaction induces differentiation both pre-synaptically and post-synaptically (Figure 4). The mechanisms have not been completely characterized in mammals, but apparently presynaptic differentiation is mediated by the scaffolding proteins liprin-α (Serra-Pagès et al., 1998; Xie et al., 2020). Liprin-α proteins participate in synaptic scaffolding and differentiation (Spangler and Hoogenraad, 2007), and they were the first intracellular proteins shown to interact with D1 and D2 domains of LAR-RPTPs through their C-terminal SAM (sterile α-motif) domains (Pulido et al., 1995; Spangler and Hoogenraad, 2007; Xie et al., 2020). Liprin interaction with D1 and D2 domains of LAR-RPTPs promotes presynaptic differentiation (Kaufmann et al., 2002; Dunah et al., 2005; Han et al., 2018), possibly through liprin-α coupling with synaptic proteins such as RIM1α and ELKS/ERC, which integrates a molecular scaffold in the active zone that mediates neurotransmitter release (Schoch et al., 2002; Ko et al., 2003b). Furthermore, liprin-α regulates the location of LAR-RPTPs by promoting membrane clustering, which also inhibits LAR-RPTPs catalytic activity as mentioned previously (Serra-Pagès et al., 1998; Bomkamp et al., 2019; Xie et al., 2020).

### Synaptic Partners Common for All LAR-RPTPs

The first molecule discovered to interact *trans*-synaptically with all three LAR-RPTPs was NGL-3, a synaptic adhesion molecule involved in synaptic formation and neurotransmission (Kwon et al., 2010; Woo et al., 2009). Interaction between PTPRF and PTPRS with NGL-3 induces pre and postsynaptic differentiation when contacting axons and dendrites respectively, forming a *trans*-synaptic complex that induces bidirectional excitatory synaptic formation (Kwon et al., 2010; Woo et al., 2009). Presynaptic PTPRF and PTPRS interact with postsynaptic NGL-3 to promote excitatory postsynaptic differentiation, which is mediated by the direct interaction between NGL-3 and PSD-95 (Kwon et al., 2010). PTPRD also interacts with NGL-3 to promote excitatory differentiation, but in a unidirectional manner. Therefore, PTPRD appears to be the only LAR-RPTP unable to induce PSD-95 recruitment and postsynaptic differentiation when interacting with NGL-3 (Kwon et al., 2010). All three LAR-RPTPs also interact with SALM3 and SALM5, members of the SALM family of cell adhesion-like proteins that modulate differentiation, maintenance, and plasticity of the synapse (Ko et al., 2006), and their interaction with LAR-RPTPs promotes excitatory synapse development (Choi et al., 2016; Li et al., 2015; Lie et al., 2016). The interaction between LAR-RPTPs and SALM3 synaptic differentiation is apparently induced in a bidirectional manner, since SALM3 binding to each LAR-RPTP recruits excitatory presynaptic proteins (Li et al., 2015), and aggregation of SALM3 on dendritic surfaces induces clustering of PSD-95 (Mah et al., 2010). Interaction between SALM3 and LAR-RPTPs can be inhibited by the *cis*-interaction of SALM3 and SALM4, and therefore this interaction induces an inhibition of the SALM3-dependent excitatory presynaptic differentiation (Lie et al., 2016). On the other hand, the interaction between LAR-RPTPs and SALM5 induces unidirectional presynaptic differentiation due to lack of a PDZ-binding motif necessary to interact with PSD-95 in the postsynaptic (Choi et al., 2016; Goto-Ito et al., 2018; Mah et al., 2010).

### PTPRF Synaptic Partners

Besides NGL-3 and SALM3/5, a PTPRF *cis*-interaction with netrin-G1 has been observed, which can also induce excitatory differentiation in a unidirectional manner. Presynaptic netrin-G1 interacts with postsynaptic netrin-G ligand-1 (NGL-1), and simultaneously directly interacts with adjacent PTPRF to shape pre-synaptic excitatory synapsis (Song et al., 2013). However, it remains to be confirmed if this interaction also occurs *in vivo*.

### PTPRD Synaptic Partners

PTPRD is the only LAR-RPTP whose expression has been observed in both excitatory and inhibitory synapses, where its interaction with NGL-3, SALM3/5, IL1RAPL1 and IL1RAcP induce excitatory differentiation, while its interaction with Slitrk1, Slitrk2 and Slitrk3 induce inhibitory synaptic differentiation (Goto-Ito et al., 2018; Kwon et al., 2010; Li et al., 2015; Lin et al., 2018; Park et al., 2020; Takahashi et al., 2012; Valnegri et al., 2011; Yim et al., 2013; Yoshida et al., 2011; Yoshida et al., 2012). Axonal PTPRD induces excitatory synaptic development bidirectionally through the interaction with its postsynaptic ligand IL1RAPL1 (Valnegri et al., 2011; Yoshida et al., 2011; Park et al., 2020). PTPRD-IL1RAPL1 interaction recruits RhoGTPase-activating protein 2 (RhoGAP2) in the post-synaptic density which promotes a signaling pathway that favors excitatory synapse development and dendritic spine formation (Valnegri et al., 2011; Park et al., 2020). Also, the postsynaptic excitatory differentiation induced by the interaction of PTPRD and IL1RAPL1 depend on the modulation of c-*Jun* terminal kinase (JNK) signaling pathway, since PTPRD activates IL1RAPL1, inducing JNK activation, which phosphorylates PSD-95 and promotes its synaptic clustering (Pavlowsky et al., 2010; Park et al., 2020). However, whether RhoGAP2 participates in JNK-mediated synaptic differentiation has not been clarified. Also, PTPRD interacts with a brain isoform of IL1RAcP, a protein essential for immune response, which promotes excitatory synaptic differentiation bidirectionally (Yoshida et al., 2012; Yamagata et al., 2015). Although IL1RAcP has homology to IL1RAPL1, it is not known whether they share a common mechanism for PTPRD-mediated synaptic differentiation. Slitrk1/2/3 are also synaptic partners for PTPRD, and their interaction induces inhibitory synapse development in a unidirectional manner, since the complex induces a presynaptic GABAergic synapse differentiation, without necessarily shaping the postsynaptic (Takahashi et al., 2012; Yim et al., 2013).

Recently, a study has identified Neuroligin3 (NLGN3) as a new ligand for PTPRD. This interaction induces excitatory or inhibitory post-synaptic differentiation depending on micro-exon meB inclusion (Yoshida et al., 2021). Furthermore, it was also observed that PTPRD and NLGN3 mediate social behaviors such as social preference and negative social response and regulate excitatory/inhibitory synaptic differentiation (Yoshida et al., 2021), highlighting the role of PTPRD splicing and its isoform interactions in excitatory/inhibitory balance.

### PTPRS Synaptic Partners

TrkC is one of the major postsynaptic partners for PTPRS, and their *trans*-synaptic interaction generates the development of excitatory synapse in a bidirectional manner (Takahashi et al., 2011; Han et al., 2018), where this complex recruits synapsin (mediated by the D2 domain of PTPRS) in the presynaptic and PSD-95 at the postsynaptic (Takahashi et al., 2011). Also, TrkC competes with HS for PTPRS interaction. HS-bound PTPRS molecules tend to assemble as oligomers at the presynaptic membrane, which inactivates its catalytic activity and promotes axon growth, whilst unbound PTPRS monomers tend to interact with TrkC to induce synaptic differentiation (Coles et al., 2011; Won et al., 2017; Han et al., 2018; Han et al., 2020a). Therefore, PTPRS interaction with both ligands and its consequent participation in each cellular process must be tightly regulated, suggesting the existence of an undefined developmental mechanism to switch PTPRS functions. When oligomeric PTPRS interacts with HS, it forms a *cis-*complexes with glypican-4 (GPC-4) with high affinity (Ko et al., 2015). GPC-4 also interacts with the postsynaptic molecule LRRTM4, which induces a bidirectional excitatory synaptic differentiation mediated by PTPRS-GPC-4-LRRTM4 complex formation, and where presynaptic differentiation depends on the PTPRS catalytic activation induced by GPC-4 binding (Ko et al., 2015; Roppongi et al., 2020). This mechanism is developmentally regulated, since PTPRS only interacts with cleaved GPC-4, and its proteolytic cleavage is reduced during postnatal development (Ko et al., 2015). On the other hand, it has been observed that LRRTM4-mediated synaptic differentiation is also dependent on PTPRS *cis* interaction with HS chains of Neurexins, where PTPRS-Neurexin-LRRTM4 and PTPRS-GPC-4-LRRTM4 complexes would coexist independently to induce the same process (Roppongi et al., 2020). However, a recent study has suggested that PTPRS *cis* interaction with HS chains of Neurexins would have an antagonistic role in synaptic differentiation. In mouse cultured hippocampal neurons, the interaction between PTPRS and HS chains of Neurexin1α inhibited excitatory post-synaptic differentiation (Han et al., 2020a). It is important to highlight that HS binding to PTPRS is mediated through Ig like domains and its affinity is highly dependent on PTPRS alternative splicing (Han et al., 2020a). Therefore, synaptic differentiation mediated by PTPRS should be carefully addressed since PTPRS splice variants might modulate differential synaptic pathways even when interacting with the same ligand. Presynaptic PTPRS interacts with Slitrks 1, 2, 4, 5 and 6 through the Ig domains to coordinate the development of the excitatory synapse in a unidirectional manner (Yim et al., 2013; Han et al., 2018). Slitrks-mediated presynaptic differentiation depends on the direct interaction between liprin-α and the PTPRS D2 domain, and also on the recruitment of the presynaptic proteins p250GAP and N-cadherin (Han et al., 2018), showing that PTPRS binding to Slitrks ligands promotes the intracellular assembly of a complex presynaptic differentiation machinery.

Since the interaction of LAR-RPTPs with their synaptic partners controls the formation of excitatory and inhibitory synapses, with profound consequences for the development of the brain, their expression must be regulated in a highly coordinated manner during neurodevelopment. Although the mechanisms involved in the cell-specific expression of LAR-RPTPs and their synaptic partners are unknown, it is possible to suggest that those neurodevelopmental disorders in which the balance between excitatory and inhibitory synapses is lost may be due at least in part to dysregulations in the mechanisms that control the expression of LAR-RPTPs (Nelson and Valakh, 2015; Parenti et al., 2020; Yoshida et al., 2021).

## LAR-RPTPs in Brain Development

Studies using LAR-RPTP knockout mice have revealed the importance of these molecules in brain development. Mice lacking the expression of each LAR-RPTP show neurodevelopmental phenotypes such as alteration in the number and size of neurons and neural precursors, altered neurogenesis in the hippocampus and brain cortex, aberrant cytoarchitecture, enhanced axon sprouting and axon targeting defects (Bernabeu et al., 2006; Takahashi and Craig, 2013; Um and Ko, 2013; Tomita et al., 2020). Accordingly, LAR-RPTPs are also implicated in developmental disorders through mechanisms such as reductions in intrinsic programmed cell death and stem cell proliferation (Uetani et al., 2009; Stewart et al., 2013).

### PTPRF

PTPRF knockout mice display increased proliferation in embryonic hippocampal neural precursor cells (NPCs) *in vitro* and in adult hippocampal NPCs *in vivo*, which is associated with an increased hippocampal neurogenesis in the adult mouse brain (Bernabeu et al., 2006), although the mechanism for PTPRF neurogenic regulation has not yet been characterized.

### PTPRD

**FIGURE 5:**
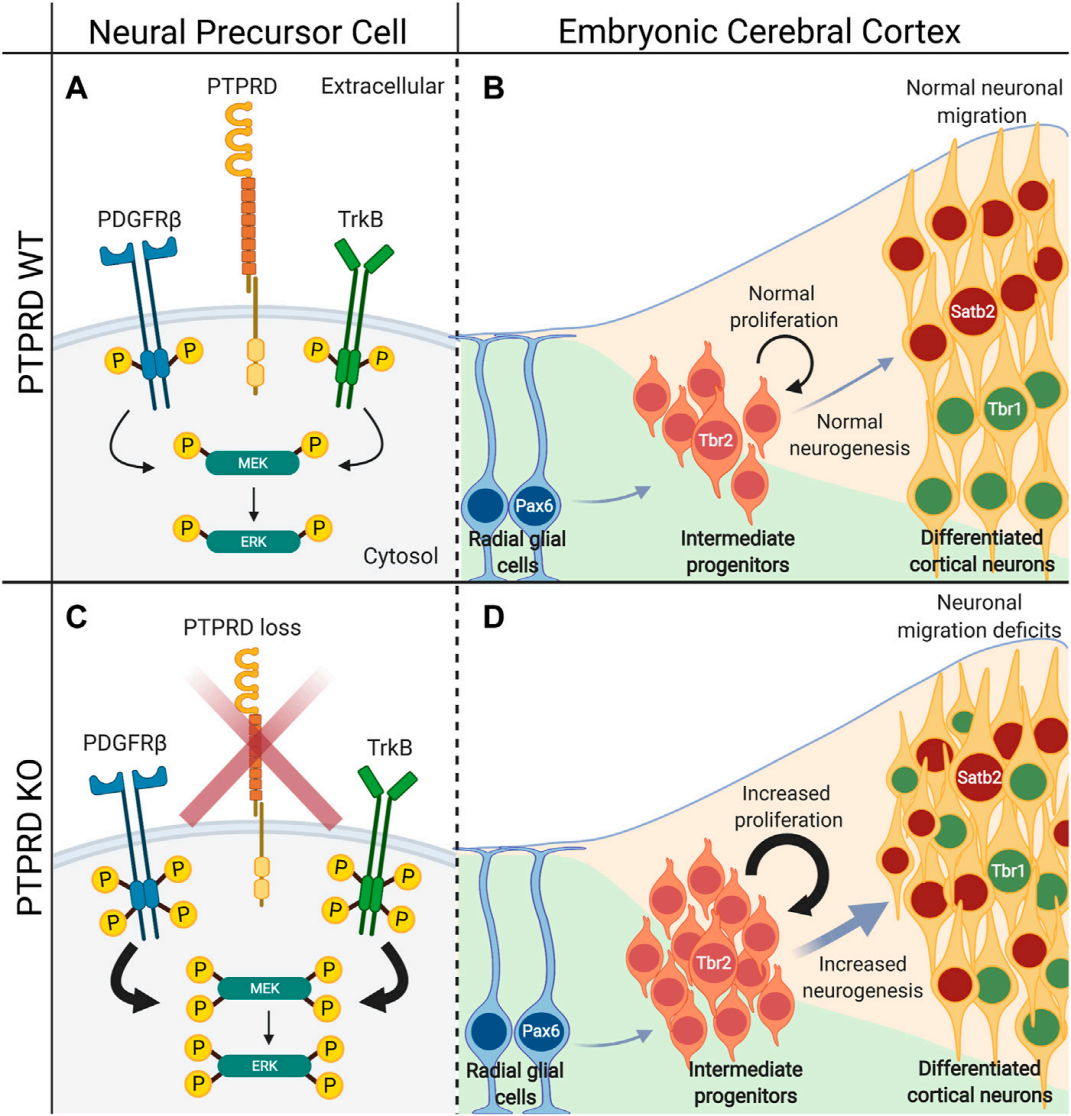
PTPRD absence induce aberrant embryonic cortical neurogenesis. **(A)** PTPRD dephosphorylates PDGFRβ and TrkB receptor tyrosine kinases to control their activity, and the activation of MEK/ERK intracellular signaling. **(B)** This induces the normal Tbr2-positive intermediate progenitor cells proliferation and neurogenesis, and the correct localization of Satb2 and Tbr1-positive neurons into the brain cortex. However **(C)** when PTPRD expression is lost, NPCs have increased phosphorylation of PDGFRβ and TrkB, which derives in the hyperactivation of the MEK/ERK intracellular signaling. **(D)** This induces an increase in Tbr2-positive intermediate progenitor cell proliferation, and consequently, aberrant increased neurogenesis and impaired positioning of Satb2 and Tbr1-positive neurons into the brain cortex.

Recently, our group found that PTPRD knockout mice have impaired neuronal differentiation and neuronal localization in the brain cortex (Tomita et al., 2020). First, we observed that the PTPRD heterozygous or homozygous knockout mice display increased neuronal intermediate progenitor cells (Tbr2 positive) without changing radial glial cell numbers (Pax6 positive). Also, the intermediate progenitor cells are highly proliferative, resulting in an increased number of differentiated cortical neurons and mislocalization of Satb2 and Tbr1-positive neurons into their corresponding cortical layers. These effects seem to be dependent on PTPRD catalytic activity and its interaction with the neurogenesis-associated receptor tyrosine kinases TrkB and PDGFRβ. Indeed, PTPRD deletion increases phosphorylation of both receptors and downstream kinase effectors MEK1 and ERK1/2 (Figure 5). Furthermore, *in vitro* inhibition of this signaling pathway by either pharmacological or RNAi strategies rescues neurogenic impairments observed in the absence of PTPRD (Tomita et al., 2020), providing a direct link between PTPRD mutations and neurodevelopmental disorders. However, it is important to highlight that considering differential expression of PTPRD in a variety of neural cell populations and elucidating how PTPRD controls receptor tyrosine kinases will be key for determining the mechanisms by which PTPRD regulates these biological processes during embryonic cortical development.

### PTPRS

In the spinal cord, it has been observed that PTPRF and PTPRS interaction with CSPGs inhibits NPC growth, attachment, survival, proliferation, and oligodendrocyte differentiation from NPCs (Dyck et al., 2015). Although the participation of PTPRF and PTPRS in NPC biology and neurodevelopment have not yet been characterized in the brain, NPCs cultured from the subventricular zone of PTPRS knockout mice show increased cellular migration from the neurosphere center, (Kirkham et al., 2006), suggesting a role of PTPRS in controlling NPC migration. In addition, mice lacking PTPRS expression show several neurological defects such as reduced number of choline acetyl transferase (ChAT)-positive neurons, slower nerve conduction velocity as a consequence of smaller myelinated fibers and hypomyelination, accompanied by behavioral alterations such as spastic movements, tremor and abnormal limb flexion among others. This suggests a role for PTPRS in the differentiation and/or development of cholinergic neurons and glial cells (Wallace et al., 1999). In addition, these mice showed deficits in the formation of the pituitary, with an elongated intermediate lobe, and smaller anterior and posterior lobes. This is accompanied by an overall decrease in brain size, a smaller olfactory bulb, and a severe depletion of luteinizing hormone-releasing-positive cells associated to a reduced size of the hypothalamus (Elchebly et al., 1999), suggesting a role for PTPRS in the development of neural cells in some brain areas including the hypothalamus-pituitary axis.

The evidence summarized in this section shows that LAR-RPTPs participate in the regulation of NPCs proliferation, neural differentiation, and neuronal migration, showing an important role in brain development, and their potential participation in the etiology of some neurodevelopmental disorders due to their impaired expression. However, the role of LAR-RPTPs in neurodevelopment remains an understudied field since LAR-RPTP ligands and substrates that participate in brain development have not been fully characterized.

## Role of LAR-RPTPs in Neurological Disorders

Many of the pathologies associated with LAR-RPTPs are systemic dysfunctions including cancer, metabolic diseases, and ulcerative colitis, among others (Chagnon et al., 2004; Muise et al., 2007; Muise and Rotin, 2008). However, there is also evidence that implicates LAR-RPTPs and its synaptic interacting proteins in neurological disorders. Here we will discuss briefly the main human neurological conditions induced by LAR-RPTPs dysfunction and the related phenotypes observed in LAR-RPTPs knockout mice, which are summarized in Table 1.

**TABLE 1:** LAR-RPTPs knock out models and their phenotypes. Different animal models lacking LAR-RPTPs expression and its induced phenotype have been summarized.

LAR-RPTP(s)	Model	Cellular phenotype	Behavioral impairments	References
**PTPRF**	PTPRF KO	↓ focal adhesions, adhesion to ECM and neurite growth	Spatial learning impairments.	Bernabeu et al. (2006), Dunah et al. (2005), Kolkman et al. (2004), Sarhan et al. (2016b), Van der Zee et al. (2003), Van Lieshout et al. (2001), Yeo et al. (1997)
		↑ NPCs proliferation and neurogenesis in the hippocampus	Increased nocturnal activity	
		↓ number and size of cholinergic neurons		
		↓ hippocampal cholinergic innervation		
		↓ regeneration and collateral axonal sprouting		
**PTPRD**	PTPRD KO	↓ dendritic branching, length, and thickness	Impaired spatial learning	Drgonova and Walther (2015), Nakamura et al. (2017), Tomita et al. (2020), Uetani et al. (2000), Uetani et al. (2006), Zhu et al. (2015)
		↑ axon degeneration	Impaired memory	
		↑ hippocampal LTP	Impaired locomotive behaviors	
		↑ cortical neurogenesis	Motor deficits	
		↑ neuronal differentiation		
		↓ cortical neuronal migration		
	PTPRD cKO (Emx1-Cre)	↓ synaptic development	Hyperactivity	Park et al. (2020)
		↓ excitatory synaptic transmission	REM sleep disturbances	
	PTPRD cKO (Nestin-Cre)	Normal number of excitatory and inhibitory synapses		Han et al. (2020c)
		Normal synaptic transmission		
		Normal vesicle tethering		
		Normal neurotransmitters release at postsynaptic targets		
	**PTPRS**	PTPRS KO	↑ dendritic density and length	Increased recognition memory. Spastic movements, tremor and ataxic gait.	Coles et al. (2011), Elchebly et al. (1999), Fry et al. (2009), Horn et al. (2012), Kirkham et al. (2006), Lang et al. (2015), Meathrel et al. (2002), McLean et al. (2002), Shen et al. (2009), Sapieha et al. (2005), Thompson et al. (2003), Wallace et al. (1999)
			↑ axon growth	Abnormal limb flexion. Defective proprioception	
↑ axonal elongation rate					
↑ growth cone elongation					
↑ axon regeneration					
↑ axon collateral branching					
↑ mEPSC frequency and paired-pulse facilitation					
↓ LTP in the hippocampus					
↑ NPCs migration					
↓ ChAT-positive neurons					
↓ myelination					
↓ luteinizing hormone-releasing cells					
PTPRS cKO (Emx1-Cre)		↓ NMDAR-dependent synaptic transmission and plasticity in the hippocampus	Deficits recognition memory	Kim et al. (2020)
			Impairment in social novelty	

### PTPRF

Decreased expression of PTPRF has been observed in induced pluripotent stem cells (iPSCs) obtained from Huntington’s disease (HD) patients, which could have a role in some pathological features of this diseases, such as neural dysfunction and cell death (The HD iPSC Consortium, 2012). In this study, it was observed that HD iPSCs showed significantly less binding to the actin cytoskeleton compared to control iPSCs, in addition to showing cell-cell adhesion deficits in culture. The loss of PTPRF phosphatase activity has previously been associated with a decrease in focal adhesions (Sarhan et al., 2016a), which could affect the interaction of the actin cytoskeleton with the extracellular proteins (Wehrle-Haller, 2012). Therefore, the decrease in actin stability and increased actin dynamics in HD iPSC could be the result of reduced number of focal adhesions. Although it is not known if the reduced expression of PTPRF in HD could have a causative role for this disorder, it might be contributing to the progression of the disease by impairing the ability of neural cells to survive and differentiate in a correct way as a result of alterations in cell adhesion. In neuronal ceroid lipofuscinoses (NCL), a neurodegenerative disorder characterized by blindness, dementia and cortical atrophy, the absence of the soluble lysosomal palmitoylthioesterases Cln1 and Cln5, induces a significant reduction in the expression of PTRPF. Cortical transcription profiles of Cln1 and Cln5 deficient animals revealed profound alterations in genes related to protein phosphorylation that affect the dynamics of the cytoskeleton and neuronal growth cones. Intermediary proteins of these signaling pathways also showed an altered subcellular distribution in culture and brain tissue assays. Although the impairment of genes that control the balance of cytoskeletal dynamics such as PTPRF are not the only cause of NCL, they prove to be important components that contribute to the pathogenesis behind neurodegeneration (von Schantz et al., 2008). Also, in immune-mediated demyelinating diseases, PTPRF expression is upregulated in exosomes obtained from patient cerebrospinal fluid, which is associated with the development of demyelinating diseases, and has been proposed as a biomarker for its early diagnosis (He et al., 2019).

PTPRF deficient mice have been developed to study PTPRF dysfunction. It has been observed that PTPRF knockout animals have a reduced number of cholinergic neurons in the forebrain and a reduced innervation of this cells to the hippocampus, an effect that was also observed in mice lacking PTPRF intracellular domain (Yeo et al., 1997; Van Lieshout et al., 2001). PTPRF deficiency also leads to a behavioral phenotype including induced spatial learning impairments and hyperactivity (Kolkman et al., 2004). Bernabeu and colleagues (2006) also showed that mice lacking PTPRF expression have an increased neurogenesis and increased NPC proliferation in the adult hippocampus (Bernabeu et al., 2006), suggesting a differential role for PTPRF in the forebrain and the hippocampus. Also, PTPRF knockout mice show reduced regenerative and collateral axonal sprouting in peripheral nerves and the forebrain (Van der Zee et al., 2003), and developmental impairments in the mammary gland cells, urogenital malformations, and an impaired craniofacial morphogenesis (Schaapveld et al., 1997; Uetani et al., 2009; Stewart et al., 2013). These animal model studies suggest an important role for PTPRF in the basal forebrain cholinergic signaling, hippocampal neurogenesis, axonal regeneration, and stem cell differentiation.

### PTPRD

*PTPRD* mutations have been directly related to neurological disorders such as restless legs syndrome (RLS) (Schormair et al., 2008; Winkelmann et al., 2011; Yang et al., 2011), obsessive-compulsive disorder (OCD) (Mattheisen et al., 2015; Gazzellone et al., 2016), autism spectrum disorders (ASDs) (Pinto et al., 2010; Levy et al., 2011; Gai et al., 2012; Liu et al., 2016; Ji et al., 2021), attention deficit and hyperactivity disorder (ADHD) (Elia et al., 2010), schizophrenia (Li et al., 2018), intellectual disabilities (Choucair et al., 2015), bipolar disorder (Malhotra et al., 2011), addictions (Drgon et al., 2010; Johnson et al., 2008; Uhl et al., 2008a; Uhl et al., 2008b; Uhl et al., 2010), and tauopathies such as Alzheimer’s disease (AD) (Chibnik et al., 2018). The main *PTPRD* genetic alterations observed in patients have been summarized in Table 2.

**TABLE 2:** Brain disorders induced by *PTPRD* mutations. Mutations in *PTPRD* have been associated with the development of several brain disorders such as intellectual disabilities, ASD, ADHD, OCD, schizophrenia, RLS, AD, and drug addictions. *PTPRD* genetic variations and its genomic location observed for each brain disorder are summarized.

Disorder	Genetic variation	Location	References
Intellectual	CNV - Homozygous Deletion	9p22.3	Choucair et al. (2015)
Disability			
ASD	CNV - Hemizygous Deletion	n/a	Gai et al. (2012), Liu et al. (2016)
ADHD	CNV - Hemizygous Deletion	Start = 9,084,805, end = 9,178,865	Elia et al. (2010)
		Start = 9,168,137, end = 10,067,180	
		Start = 9,985,938, end = 10,020,458	
OCD	CNV - Duplication	9p24.1	Gazzellone et al. (2016)
	SNP	1.28 Mb from the 5′ end of *PTPRD*	Mattheisen et al. (2015)
Schizophrenia	SNP	n/a	Li et al. (2018)
RLS	SNP	5′UTR, rs1975197, bp = 8,836,955	Schormair et al. (2008)
		5′UTR, rs4626664, bp = 9,251,737	
AD	SNP	rs560380, bp = 9,112,698	Chibnik et al. (2018)
Drug Addiction	SNP	rs12001948	Uhl et al. (2008a), Uhl, et al. (2008b)
		rs7854145	
		rs2221184	
		rs10511496	

One of the brain disorders in which *PTPRD* mutations have an important functional impact is RLS, also known as Willis–Ekbom disorder, which is characterized by symptoms including an urge to move, usually accompanied by uncomfortable sensations in the lower limbs (Schormair et al., 2008). The etiology has not yet been determined but may be related to dopaminergic and iron imbalance (Allen, 2004). The relationship between PTPRD and RLS has been established by genome wide association studies (GWAS), where single nucleotide polymorphisms (SNPs) frequently lead to reduced *PTPRD* mRNA expression (Drgonova and Walther, 2015; Hawrylycz et al., 2012). It is important to note that RLS is effectively treated with dopamine agonists and GABA analogs (Nagandla and De, 2013), although whether *PTPRD* mutations impair dopaminergic neurotransmission remains unclear.

In OCD, a psychiatric disorder characterized by compulsive behaviors that patients perform in response to obsessive thoughts, *PTPRD* variants have been associated with this disorder in two GWAS studies. These include a SNP 1.28 Mb from 5′ end of *PTPRD* gene (Mattheisen et al., 2015), and copy number variants (CNVs) arising from a duplication of 1.5 Mb at 9p24.1 (Gazzellone et al., 2016). Although there is no information on how these variants affect the expression of PTPRD, mouse models deficient for PTPRD show impairments in learning and memory tasks (Uetani et al., 2000), which is relevant for OCD, since memory impairments have been previously reported in OCD patients (Jaafari et al., 2013). Interestingly, *PTPRD* duplications may also contribute to the development of neurological disorders. It has been observed that duplication (71391bp) of the *PTPRD* gene at 9p23 has been related to an increased risk of suffering bipolar disorder (Malhotra et al., 2011), suggesting that increased PTPRD expression could also be involved in brain pathologies.

In brain disorders such as ASD, ADHD and intellectual disability, CNVs or mutations in the *PTPRD* gene (Elia et al., 2010; Pinto et al., 2010; Levy et al., 2011; Choucair et al., 2015) are directly associated with neurodevelopmental impairments. This is consistent with the phenotype of PTPRD knockout mice, which show impaired neuronal differentiation and disrupted cortical organization (Tomita et al., 2020). These mice also display reduced IL1RAPL1-mediated synapse formation (Yoshida et al., 2011), presumably because PTPRD knockout induces a significant reduction in IL1RAPL1 expression (Choucair et al., 2015; Park et al., 2020). Furthermore, specific mutation of the PTPRD IL1RAPL1-interacting domain induces hyperactivity and sleep disturbance, which is also observed in PTPRD conditional knockout mice (Park et al., 2020). Finally, PTPRD knockout mice show body growth retardation and spatial learning and memory impairments, which surprisingly correlates with an enhanced synaptic transmission in the hippocampus (Uetani et al., 2000).

In AD, an SNP in the *PTPRD* locus shows a mild association with disease, but significant association with accumulation of neurofibrillary tangles (Chibnik et al., 2018). This may be induced by increased levels of phosphorylated tau protein in a PTPRD dependent mechanism, since its participation in tau phosphorylation signaling pathway has been previously suggested (Mitchell et al., 2016), and impaired PTPRD catalytic activity or expression could reduce tau dephosphorylation, although this hypothesis has not yet been tested.

### PTPRS

Although no brain pathologies have been causally linked to *PTPRS* mutations or impaired function, in a rat model of Amyotrophic lateral sclerosis (ALS), reduced neuronal expression of PTPRS has been observed. This may inhibit axonal regeneration and favor glial scar formation through its interaction with CSPGs (Ohtake and Li, 2015; Shijo et al., 2018). Also, PTPRS has recently been considered as a potential target for AD treatment (Gu et al., 2016). PTPRS interacts with amyloid precursor protein (APP) in the brain, and by knocking PTPRS expression, the affinity between β-secretase and APP can be reduced, decreasing Aβ extracellular accumulation and inhibiting tau aggregation without affecting β-secretase enzymatic activity. Moreover, PTPRS knockout rescues behavioral impairments observed in an AD mice model (Gu et al., 2016). This suggests PTPRS as a potential target for selective pharmacological intervention in AD.

As mentioned earlier, mice lacking PTPRS expression show body growth retardation, an abnormal physiology of the posterior pituitary, reduced hormone-releasing cells in the hypothalamus and reduced cholinergic neurons in the forebrain, which associates with neurological impairments such as spastic movements, tremor, ataxic gait, abnormal limb flexion and defective proprioception (Elchebly et al., 1999; Wallace et al., 1999). Moreover, PTPRS knockout mouse show an atypical hippocampus morphology, and a reduced thickness in the corpus callosum and brain cortex, which is suggested to be a consequence of a delayed neurodevelopment and an abnormal NPCs biology (Meathrel et al., 2002; Kirkham et al., 2006). On the other hand, Horn and colleagues (2012) have observed that PTRPS knockout mice show increased dendritic spine density and length, increased frequency of mEPSC, a greater paired-pulse facilitation, and a reduced long-term potentiation in the hippocampus, which correlates with an increased recognition memory, assessed by the novel object recognition test (Horn et al., 2012).

### Other Implications

The spectrum of brain disorders involving LAR-RPTPs becomes wider when we consider that their functions in neural cells depend on their interactions. LAR-RPTP synaptic partners such as Slitrks, TrkC and IL1RAPL1, have also been implicated in neurological disorders (Um and Ko, 2013). For instance, Slitrk mutations have been associated with Tourette’s syndrome, trichotillomania, schizophrenia, ASD and epilepsy (Piton et al., 2011; Wu et al., 2013). TrkC mutations have been found in patients with AD, depression, bipolar disorder, and ADHD (Otnæss et al., 2009; Amador-Arjona et al., 2010). IL1RAPL1 has been related to a form of X-linked intellectual disability (Behnecke et al., 2011; Youngs et al., 2012). Hence, the links between LAR-RPTP impairments and the development of neurological disorders, combined with the evidence associating impairments in the LAR-RPTPs synaptic partners with neuronal pathologies, open a wide spectrum of brain disorders where LAR-RPTPs may be either directly or indirectly related. Finally, it is important to highlight that, impairments in the regulation of LAR-RPTP alternative splicing also induce psychiatric disorders. Neuronal micro-exons in LAR-RPTPs are dysregulated in brain samples from patients with ASD, suggesting that disruption of a coordinated program of LAR-RPTP splicing events may lead to neurodevelopmental pathologies (Irimia et al., 2014; Quesnel-Vallières et al., 2015). Therefore, the manipulation of alternative splicing machinery could be used as a therapeutical approach to restore the impaired neural development and the inhibitory/excitatory imbalance observed in neurodevelopmental pathologies such as ASD (Nelson and Valakh, 2015; Parenti et al., 2020; Yoshida et al., 2021).

In summary, LAR-RPTPs are implicated in a variety of neurodevelopmental and neurological dysfunctions, and their mutations and impaired expression is associated with cognitive impairment to dementia. Further studies are necessary to understand how LAR-RPTP genetic variants can contribute to generate diverse brain pathologies. Considering that LAR-RPTPs encode extracellular interaction and intracellular catalytic and regulatory domains, variants in these regions may have diverse impact on neurobiology. Moreover, LAR-RPTPs alternative splicing variants are dependent on extremely short sequences (micro-exons), and SNPs in these critical regions could have profound consequences on LAR-RPTP neurological functions. For example, in the case of LAR-RPTP contributions to synaptic differentiation and function, even subtle genetic alterations could impair interactions with synaptic interaction partners, directly affecting the number of excitatory or inhibitory synapses. Thus, LAR-RPTP genetic variants may contribute in myriad ways to neurological processes that are disrupted in various neuropsychiatric disorders.

## Concluding Remarks

**FIGURE 6:**
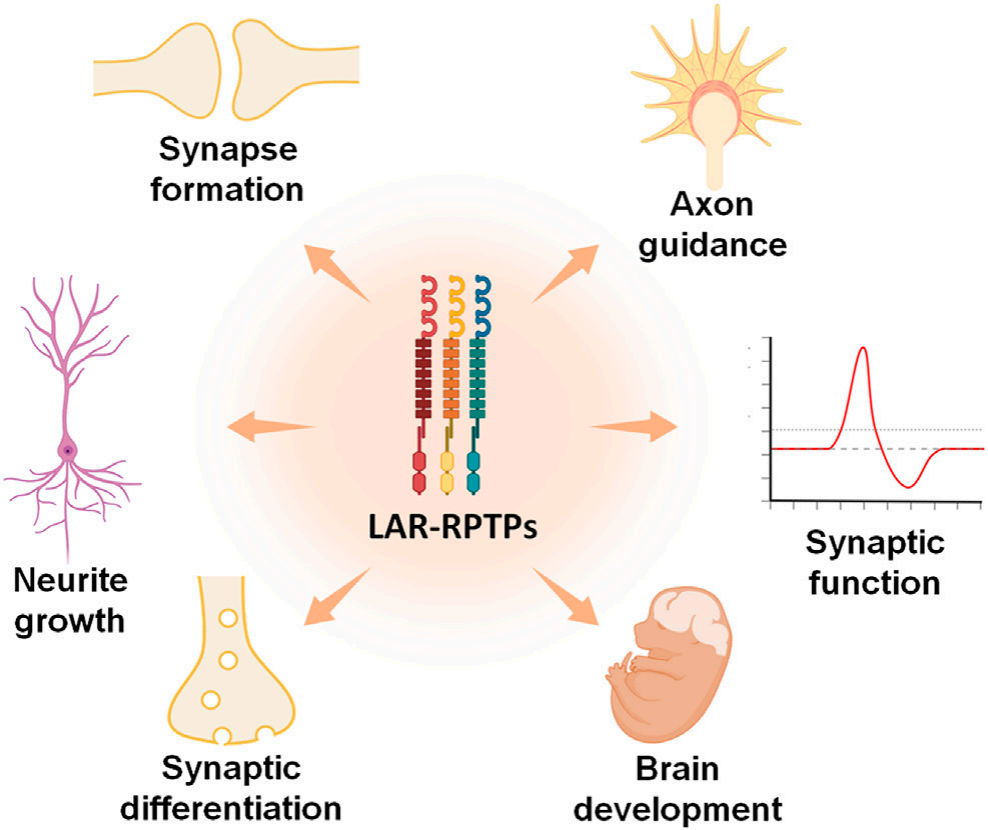
LAR-RPTPs participate in several neural functions. All three LAR-RPTPs are implicated in various functions involved in the biology of neurons, such as neurite and axon growth, axon guidance, synaptic formation and differentiation, synaptic functions, and brain development.

All the evidence summarized here confirms an important neurological role for LAR-RPTPs in health and disease. Although LAR-RPTP functions as cell adhesion molecules are well established in neurobiology, there are several lines of evidence that describes their phosphatase activity as an important modulator of neurite growth, axon guidance, synapse formation and differentiation, synaptic function, and brain development (Figure 6).

However, it is important to note that the functions of the different LAR-RPTPs in the brain are not yet fully understood, which has been illustrated by the latest research demonstrating phenotypes different from those previously observed (Han et al., 2020c; Sclip and Südhof, 2020). Given the many processes in which LAR-RPTPs participate, their expression and function must be tightly regulated. Therefore, it is important to consider all the variables that could alter LAR-RPTPs when studying them. Some of these variables are the stage of development and the cell type; in the developing mouse brain, PTPRF expression is dramatically reduced as neuroblasts differentiate and migrate (Schaapveld et al., 1998). On the other hand, PTPRD and PTPRS are highly expressed as neural cells differentiate (Schaapveld et al., 1998), but PTPRS expression is higher in the embryonic nervous system, with a parallel expression pattern to PTPRD (Chagnon et al., 2004). Also, the PTPRD isoform switch from including meA3 to including meA9 between E18.5 and P4 (Wamsley et al., 2018).

Moreover, PTPRD meB has been reported to be highly included at embryonic brain samples (E11.5), suggesting that the PTPRD *trans*-synaptic interactions promoted by meB inclusion may have a role in synaptogenesis during brain development (Yoshida et al., 2011; Yamagata et al., 2015). On the other hand, it has been recently reported that three PTPRD micro-exons that code for meA and meB are differentially included between inhibitory and excitatory neurons, suggesting that the alternative splicing of meA/B is finely tuned across neuronal cell types (Parada et al., 2021). Thus, LAR-RPTPs differential expression pattern suggests that they could exert different roles in the developing brain cells and in their function. By considering LAR-RPTPs regional and temporal expression patterns, we could study their physiological phenotype more specifically.

The evidence summarized here evidence that LAR-RPTPs are not only important molecules for shaping the synapse, but also for brain development and for its physiological functions. As a result, alterations in LAR-RPTP expression and function are associated with different brain disorders. Indeed, the diverse neurological processes in which LAR-RPTPs participate highlights the importance of studying the role of specific LAR-RPTP functions such as ligand binding and/or phosphatase activity, and how these are disrupted in brain disorders. Thus, elaborating approaches to modulate specific LAR-RPTP functions could be an important therapeutic strategy for several brain disorders.
